# Modeling the temporal dynamics of the gut microbial community in adults and infants

**DOI:** 10.1371/journal.pcbi.1006960

**Published:** 2019-06-27

**Authors:** Liat Shenhav, Ori Furman, Leah Briscoe, Mike Thompson, Justin D. Silverman, Itzhak Mizrahi, Eran Halperin

**Affiliations:** 1 Department of Computer Science, University of California Los Angeles, Los Angeles, California, United States of America; 2 Life Sciences, Ben Gurion University, Be’er Sheva, Israel; 3 Department of Human Genetics, University of California Los Angeles, Los Angeles, California, United States of America; 4 Center for Genomic and Computational Biology, Duke University, Durham, North Carolina, United States of America; 5 Department of Computational Medicine, University of California Los Angeles, Los Angeles, California, United States of America; 6 Department of Anesthesiology and Perioperative Medicine, University of California Los Angeles, Los Angeles, California, United States of America; NYU, UNITED STATES

## Abstract

Given the highly dynamic and complex nature of the human gut microbial community, the ability to identify and predict time-dependent compositional patterns of microbes is crucial to our understanding of the structure and functions of this ecosystem. One factor that could affect such time-dependent patterns is microbial interactions, wherein community composition at a given time point affects the microbial composition at a later time point. However, the field has not yet settled on the degree of this effect. Specifically, it has been recently suggested that only a minority of taxa depend on the microbial composition in earlier times. To address the issue of identifying and predicting temporal microbial patterns we developed a new model, *MTV-LMM* (Microbial Temporal Variability Linear Mixed Model), a linear mixed model for the prediction of microbial community temporal dynamics. *MTV-LMM* can identify time-dependent microbes (i.e., microbes whose abundance can be predicted based on the previous microbial composition) in longitudinal studies, which can then be used to analyze the trajectory of the microbiome over time. We evaluated the performance of *MTV-LMM* on real and synthetic time series datasets, and found that *MTV-LMM* outperforms commonly used methods for microbiome time series modeling. Particularly, we demonstrate that the effect of the microbial composition in previous time points on the abundance of taxa at later time points is underestimated by a factor of at least 10 when applying previous approaches. Using *MTV-LMM*, we demonstrate that a considerable portion of the human gut microbiome, both in infants and adults, has a significant time-dependent component that can be predicted based on microbiome composition in earlier time points. This suggests that microbiome composition at a given time point is a major factor in defining future microbiome composition and that this phenomenon is considerably more common than previously reported for the human gut microbiome.

## Author summary

The ability to characterize and predict temporal trajectories of the microbial community in the human gut is crucial to our understanding of the structure and functions of this ecosystem. In this study we develop *MTV-LMM*, a method for modeling time-series microbial community data. Using *MTV-LMM* we find that in contrast to previous reports, a considerable portion of microbial taxa in both infants and adults display temporal structure that is predictable using the previous composition of the microbial community. In reaching this conclusion we have adopted a number of concepts common in statistical genetics for use with longitudinal microbiome studies. We introduce concepts such as time-explainability and the temporal kinship matrix, which we believe will be of use to other researchers studying microbial dynamics, through the framework of linear mixed models. In particular we find that the association matrix estimated by *MTV-LMM* reveals known phylogenetic relationships and that the temporal kinship matrix uncovers known temporal structure in infant microbiome and inter-individual differences in adult microbiome. Finally, we demonstrate that *MTV-LMM* significantly outperforms commonly used methods for temporal modeling of the microbiome, both in terms of its prediction accuracy as well as in its ability to identify time-dependent taxa.

## Introduction

There is increasing recognition that the human gut microbiome is a contributor to many aspects of human physiology and health including obesity, non-alcoholic fatty liver disease, inflammatory diseases, cancer, metabolic diseases, aging, and neurodegenerative disorders [1–14]. This suggests that the human gut microbiome may play important roles in the diagnosis, treatment, and ultimately prevention of human disease. These applications require an understanding of the temporal variability of the microbiota over the lifespan of an individual particularly since we now recognize that our microbiota is highly dynamic, and that the mechanisms underlying these changes are linked to ecological resilience and host health [15–17].

Due to the lack of data and insufficient methodology, we currently have major gaps in our understanding of fundamental mechanisms related to the temporal behavior of the microbiome. Critically, we currently do not have a clear characterization of how and why our gut microbiome varies in time, and whether these dynamics are consistent across humans. It is also unclear whether we can define ‘stable’ or ‘healthy’ dynamics as opposed to ‘abnormal’ or ‘unhealthy’ dynamics, which could potentially reflect an underlying health condition or an environmental factor affecting the individual, such as antibiotics exposure or diet. Moreover, there is no consensus as to whether the gut microbial community structure varies continuously or jumps between discrete community states, and whether or not these states are shared across individuals [18, 19]. Notably, recent work [20] suggests that the human gut microbiome composition is dominated by environmental factors rather than by host genetics, emphasizing the dynamic nature of this ecosystem.

The need for understanding the temporal dynamics of the microbiome and its interaction with host attributes have led to a rise in longitudinal studies that record the temporal variation of microbial communities in a wide range of environments, including the human gut microbiome. These time series studies are enabling increasingly comprehensive analyses of how the microbiome changes over time, which are in turn beginning to provide insights into fundamental questions about microbiome dynamics [16, 17, 21].

One of the most fundamental questions that still remains unanswered is to what degree the microbial community in the gut is deterministically dependent on its initial composition (e.g., microbial composition at birth). More generally, it is unknown to what degree the microbial composition of the gut at a given time determines the microbial composition at a later time. Additionally, there is only preliminary evidence of the long-term effects of early life events on the gut microbial community composition, and it is currently unclear whether these long-term effects traverse through a predefined set of potential trajectories [21, 22].

To address these questions, it is important to quantify the dependency of the microbial community at a given time on past community composition [23, 24]. This task has been previously studied in theoretical settings. Specifically, the generalized Lotka-Volterra family of models infer changes in community composition through defined species-species or species-resource interaction terms, and are popular for describing internal ecological dynamics. Recently, a few methods that rely on deterministic regularized model fitting using generalized Lotka-Volterra equations have been proposed (e.g., [25–27]). Nonetheless, the importance of pure autoregressive factors (a stochastic process in which future values are a function of the weighted sum of past values) in driving gut microbial dynamics is, as yet, unclear.

Other approaches that utilize the full potential of longitudinal data, can often reveal insights about the autoregressive nature of the microbiome. These include, for example, the sparse vector autoregression (sVAR) model, (Gibbons et al. [24]), which assumes linear dynamics and is built around an autoregressive type of model, ARIMA Poisson (Ridenhour et al. [28]), which assumes log-linear dynamics and suggests modeling the read counts along time using Poisson regression, and TGP-CODA (Aijo et al. 2018 [29]), which uses a Bayesian probabilistic model that combines a multinomial distribution with Gaussian processes.

Particularly, Gibbons et al. [24], uses the sparse vector autoregression (sVAR) model to show evidence that the human gut microbial community has two dynamic regimes: autoregressive and non-autoregressive. The autoregressive regime includes taxa that are affected by the community composition at previous time points, while the non-autoregressive regime includes taxa that their appearance in a specific time is random and or does not depend on the previous time points. In this paper, we show that previous studies substantially underestimate the autoregressive component of the gut microbiome.

In order to quantify the dependency of taxa on past composition of the microbial community, we introduce Microbial community Temporal Variability Linear Mixed Model (*MTV-LMM*), a ready-to-use scalable framework that can simultaneously identify and predict the dynamics of hundreds of time-dependent taxa across multiple hosts. *MTV-LMM* is based on a linear mixed model, a heavily used tool in statistical genetics and other areas of genomics [30, 31]. Using *MTV-LMM* we introduce a novel concept we term ‘time-explainability’, which corresponds to the fraction of temporal variance explained by the microbiome composition at previous time points. Using time-explainability researchers can select the microorganisms whose abundance can be explained by the community composition at previous time points in a rigorous manner.

*MTV-LMM* has a few notable advantages. First, unlike the sVAR model and the Bayesian approach proposed by Aijo et al. [29], *MTV-LMM* models all the individual hosts simultaneously, thus leveraging the information across an entire population while adjusting for the host’s effect (e.g,. host’s genetics or environment). This provides *MTV-LMM* an increased power to detect temporal dependencies, as well as the ability to quantify the consistency of dynamics across individuals. The Poisson regression method suggested by Ridenhour et al. [28] also utilizes the information from all individuals, but does not account for the individual effects, which may result in an inflated autoregressive component. Second, *MTV-LMM* is computationally efficient, allowing it to model the dynamics of a complex ecosystem like the human gut microbiome by simultaneously evaluating the time-series of hundreds of taxa, across multiple hosts, in a timely manner. Other methods, (e.g., TGP-CODA [29], MDSINE [26] etc.) can model only a small number of taxa. Third, *MTV-LMM* can serve as a feature selection method, selecting only the taxa affected by the past composition of the microbiome. The ability to identify these time-dependent taxa is crucial when fitting a time series model to study the microbial community temporal dynamics. Finally, we demonstrate that *MTV-LMM* can serve as a standalone prediction model that outperforms commonly used models by an order of magnitude in predicting the taxa abundance.

We applied *MTV-LMM* to synthetic data, as suggested by Ajio et al. 2018 [29] as well as to three real longitudinal studies of the gut microbiome (David et al. [17], Caporaso et al. [16], and DIABIMMUNE [21]). These datasets contain longitudinal abundance data using 16S rRNA gene sequencing. Nonetheless, *MTV-LMM* is agnostic to the sequencing data type (i.e., 16s rRNA or shotgun sequencing).

Using *MTV-LMM* we find that in contrast to previous reports, a considerable portion of microbial taxa, in both infants and adults, display temporal structure that is predictable using the previous composition of the microbial community. Moreover, we show that, on average, the time-explainability is an order of magnitude larger than previously estimated for these datasets.

## Results

### A brief description of *MTV-LMM*

We begin with an informal description of the main idea and utility of *MTV-LMM*. A more comprehensive description can be found in the Methods. *MTV-LMM* is motivated by our assumption that the temporal changes in the abundance of taxa are a time-homogeneous high-order Markov process. *MTV-LMM* models the transitions of this Markov process by fitting a sequential linear mixed model (LMM) to predict the relative abundance of taxa at a given time point, given the microbial community composition at previous time points. Intuitively, the linear mixed model correlates the similarity between the microbial community composition across different time points with the similarity of the taxa abundance at the next time points. *MTV-LMM* is making use of two types of input data: (1) continuous relative abundance of focal taxa *j* at previous time points and (2) quantile-binned relative abundance of the rest of the microbial community at previous time points. The output of *MTV-LMM* is prediction of continuous relative abundance, for each taxon, at future time points.

In order to apply linear mixed models, *MTV-LMM* generates a *temporal kinship matrix*, which represents the similarity between every pair of samples across time, where a sample is a normalization of taxa abundances at a given time point for a given individual (see Methods). When predicting the abundance of taxa *j* at time *t*, the model uses both the global state of the entire microbial community in the last *q* time points, as well as the abundance of taxa *j* in the previous *p* time points. The parameters *p* and *q* are determined by the user, or can be determined using a cross-validation approach; a more formal description of their role is provided in the Methods. *MTV-LMM* has the advantage of increased power due to a low number of parameters coupled with an inherent regularization mechanism, similar in essence to the widely used ridge regularization, which provides a natural interpretation of the model.

### Model evaluation

We evaluated *MTV-LMM* by testing its accuracy in predicting the abundance of taxa at a future time point using real time series data. Such evaluation will mitigate overfitting, since the future data points are held out from the algorithm. To measure accuracy on real data, we used the squared Pearson correlation coefficient between estimated and observed relative abundance along time, per taxon. In addition we validated *MTV-LMM* using synthetic data, illustrating realistic dynamics and abundance distribution, as suggested by Aijo et al. 2018 [29]. Following [29], we evaluate the performance of the model using the ‘estimation-error’, defined to be the Euclidean distance between estimated and observed relative abundance, per time point (see Supplementary Information S1 Note).

We used real time series data from three different datasets, each composed of longitudinal abundance data. These three datasets are David et al. [17](2 adult donors—DA, DB—average 250 time points per individual), Caporaso et al. [16] (2 adult donors—M3, F4—average 231 time points per individual), and the DIABIMMUNE dataset [21] (39 infant donors—average 28 time points per individual). In these datasets, the temporal parameters *p* and *q* were estimated using a validation set, and ranged from 0 to 3. See Methods for further details.

**Fig 1:**
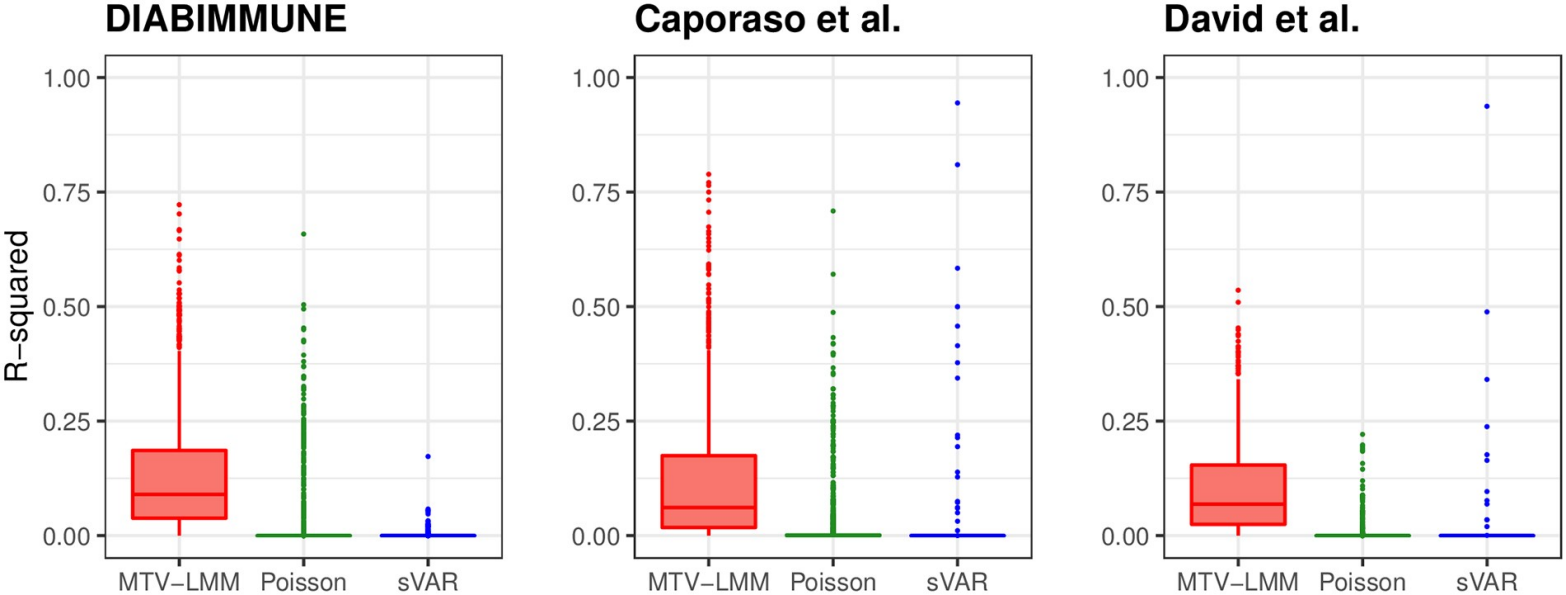
*MTV-LMM* outperforms commonly used methods in prediction accuracy (*R*^2^) and detection of autoregressive dynamics. *MTV-LMM* predictions are in red, ARIMA Poisson regression in green, and sVAR in blue.

We compared the results of *MTV-LMM* to common approaches that are widely used for temporal microbiome modeling, namely the AR(1) model (see Methods), the sparse vector autoregression model sVAR [24], the ARIMA Poisson regression [28] and TGP-CODA [29]. Overall, *MTV-LMM*’s prediction accuracy is higher than AR’s (Supplementary Information S1 Table) and significantly outperforms both the sVAR method and the Poisson regression across all datasets, using real time-series data (Fig 1). In addition, since TGP-CODA can not be fully applied to these real datasets (due to scalability limitations), we used synthetic data, considering a scenario of 200 taxa and 70 time points with realistic dynamics and abundance distribution, as suggested by the authors of this method. Similarly to the real data, *MTV-LMM* significantly outperforms all the compared methods (Supplementary Information S1 Fig).

### Inference on the estimated association matrix

**Fig 2:**
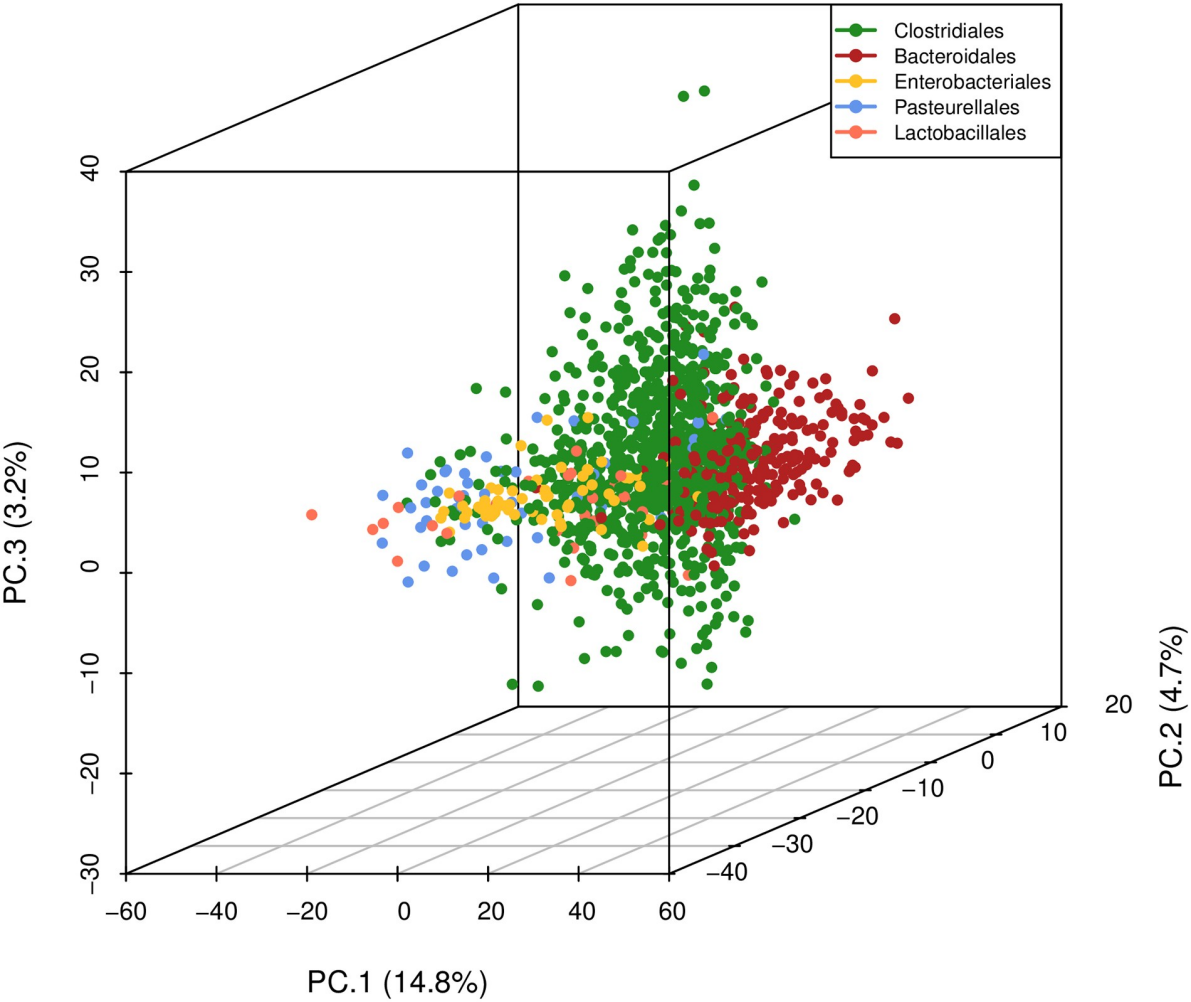
The first three principle components of the inferred association matrix recover known phylogenetic structure. Closely related species, in the DIABIMUNE dataset, have similar association patterns within the microbial community. Shown on each axis is the percentage of variance explained by each principal component for the top five orders in the data.

We applied *MTV-LMM* to the DIABIMMUNE infant dataset and estimated the species-species association matrix across all individuals, using 1440 taxa that passed a preliminary screening according to temporal presence-absence patterns (see Methods). We found that most of these effects are close to zero, implying a sparse association pattern. Next, we applied a principal component analysis (PCA) to the estimated species-species associations and found a strong phylogenetic structure (PerMANOVA P-value = 0.001) suggesting that closely related species have similar association patterns within the microbial community (Fig 2). These findings are supported by Thompson et al. [32], who suggested that ecological interactions are phylogenetically conserved, where closely related species interact with similar partners. Gomez et al. [33] tested these assumptions on a wide variety of hosts and found that generalized interactions can be evolutionary conserved.

We note that the association matrix estimated by *MTV-LMM* should be interpreted with caution since the number of possible associations is quadratic in the number of species, and it is, therefore, unfeasible to infer with high accuracy all the associations. However, we can still aggregate information across species or higher taxonomic levels to uncover global patterns of the microbial composition dynamics (e.g., principal component analysis).

### Time-explainability as a measure of the autoregressive component in the microbial community

In order to address the fundamental question regarding the gut microbiota temporal variation, we quantify its autoregressive component. Namely, we quantify to what degree the abundance of different taxa can be inferred based on the microbial community composition at previous time points. In statistical genetics, the fraction of phenotypic variance explained by genetic factors is called heritability and is typically evaluated under an LMM framework [30]. Intuitively, linear mixed models estimate heritability by measuring the correlation between the genetic similarity and the phenotypic similarity of pairs of individuals. We used *MTV-LMM* to define an analogous concept that we term *time-explainability*, which corresponds to the fraction of temporal variance explained by the microbiome composition at previous time points.

**Fig 3:**
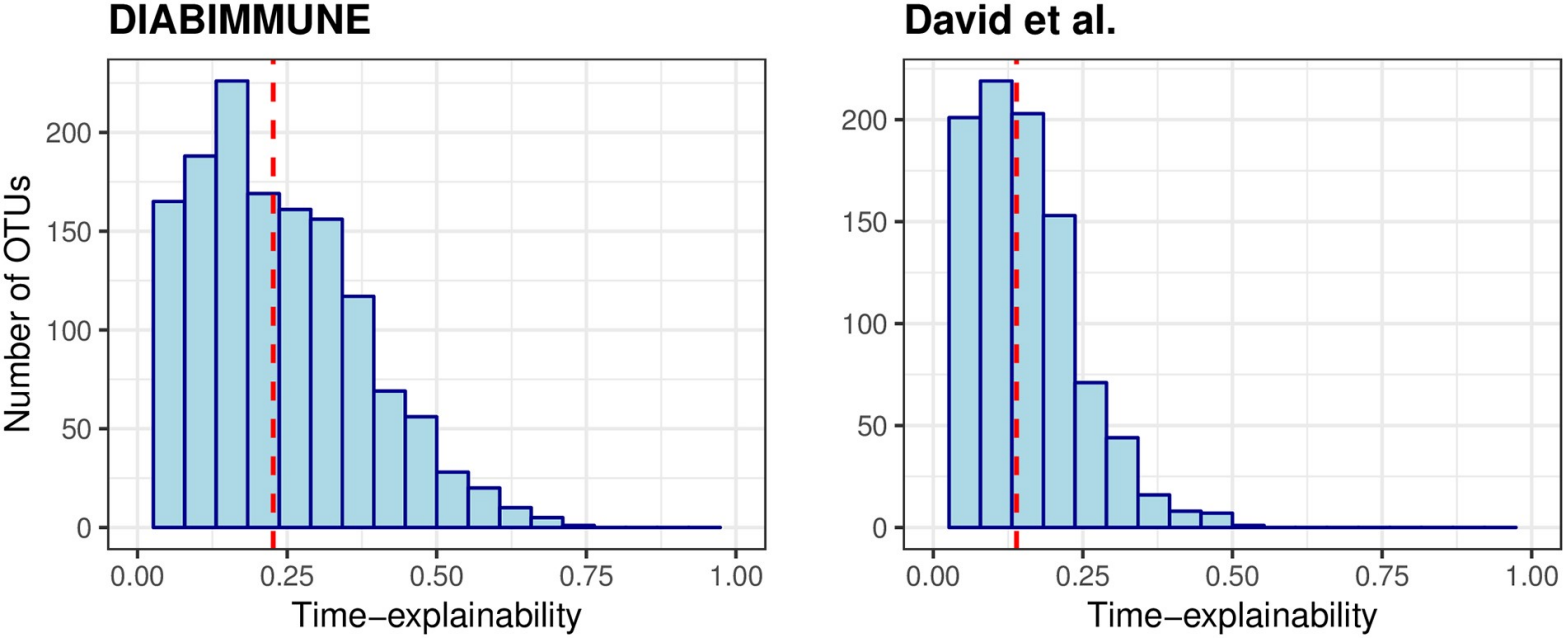
Time-explainability distribution. Time-explainability distribution in the DIABIMMUNE infant dataset (left) and David et al. adult dataset (right). The average time-explainability (denoted by a dashed line) in the DIABIMMUNE cohort is 23% and in David et al. is 14%.

In order to highlight the effect of the microbial community, we next estimated the time-explainability of taxa in each dataset, using the parameters *q* = 1, *p* = 0. The resulting model corresponds to the formula: *taxa*_*t*_ = microbiome community_(*t*−1)_ + individual effect_(*t*−1)_ + unknown effects. Of the taxa we examined, we identified a large portion of them to have a statistically significant time-explainability component across datasets. Specifically, we found that over 85% of the taxa included in the temporal kinship matrix are significantly explained by the time-explainability component, with estimated time-explainability average levels of 23% in the DIABIMMUNE infant dataset (sd = 15%), 21% in the Caporaso et al. (2011) dataset (sd = 15%) and 14% in the David el al. dataset (sd = 10%) (Fig 3, Supplementary Information S2 Fig). Notably, we found that higher time explanability is associated with higher prediction accuracy (Supplementary Information S3 Fig).

### Non-autoregressive dynamics contain phylogenetic structure

**Fig 4:**
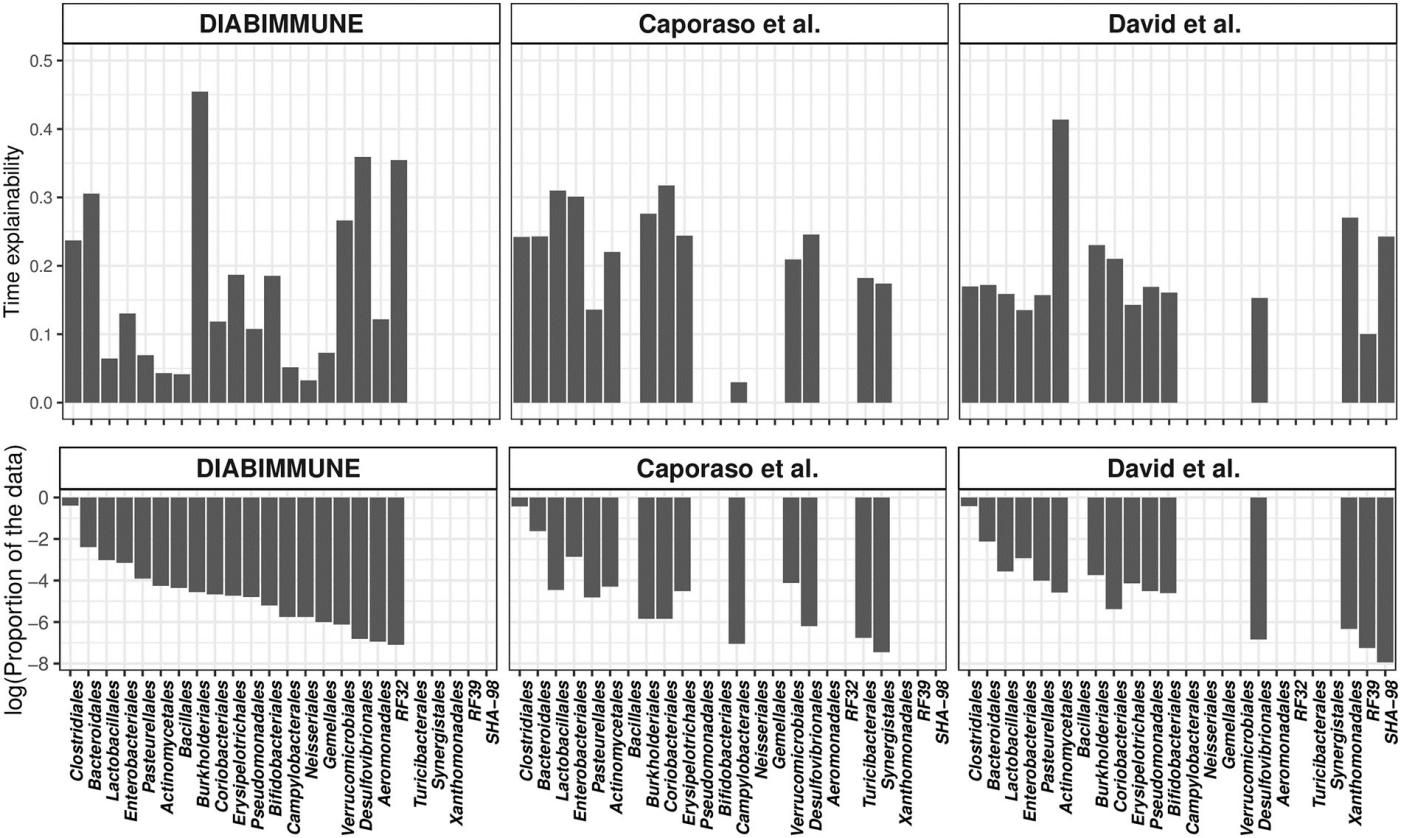
Time-explanability differs by taxonomic order across all datasets. In the top row, the y-axis is the average time-explainability (per order). In the bottom row, the y-axis is the proportion of data the order occupies (log scale). the x-axis shows orders with taxa that are autoregressive in at least one dataset.

As a secondary analysis, we aggregated the time-explainability by taxonomic order, and found that in some orders (*non-autoregressive orders*) all taxa are non-autoregressive, while in others (*mixed orders*) we observed the presence of both autoregressive and non-autoregressive taxa (Fig 4, Supplementary Information S4 Fig), where an autoregressive taxa have a statistically significant time-explainability component.

Particularly, in the DIABIMMUNE infant data set, there are 7244 taxa, divided into 55 different orders. However, the taxa recognized by *MTV-LMM* as autoregressive (1387 out of 7244) are represented in only 19 orders out of the 55. The remaining 36 orders do not include any autoregressive taxa. Unlike the autoregressive organisms, these non-autoregressive organisms carry a strong phylogenetic structure (t-test p-value < 10^−16^), that may indicate a niche/habitat filtering. This observation is consistent with the findings of Gibbons et al. [24], who found a strong phylogenetic structure in the non-autoregressive organisms in the adult microbiome.

Notably, across all datasets, there is no significant correlation between the order dominance (number of taxa in the order) and the magnitude of its time-explainability component (median Pearson *r* = 0.12). For example, in the DIABIMMUNE data set, the proportion of autoregressive taxa within the 19 mixed orders varies between 2% and 75%, where the average is approximately 20%. In the most dominant order, Clostridiales (representing 68% of the taxa), approximately 20% of the taxa are autoregressive and the average time-explainability is 23%. In the second most dominant order, Bacteroidales, approximately 35% of the taxa are autoregressive and the average time-explainability is 31%. In the Bifidobacteriales order, approximately 75% of the taxa are autoregressive, and the average time-explainability is 19% (Fig 4). We hypothesize that the large fraction of autoregressive taxa in the Bifidobacteriales order, specifically in the infants dataset, can be partially attributed to the finding made by [34], according to which some sub-species in this order appear to be specialized in the fermentation of human milk oligosaccharides and thus can be detected in infants but not in adults. This emphasizes the ability of *MTV-LMM* to identify taxa that have prominent temporal dynamics that are both habitat and host-specific.

As an example of *MTV-LMM*’s ability to differentiate autoregressive from non-autoregressive taxa within the same order, we examined Burkholderiales, a relatively rare order (less than 2% of the taxa in the data) with 76 taxa overall, where only 19 of which were recognized as autoregressive by *MTV-LMM*. Indeed, by examining the temporal behavior of each non-autoregressive taxa in this order, we witnessed abrupt changes in abundance over time, where the maximal number of consecutive time points with abundance greater than 0 is very small. On the other hand, in the autoregressive taxa, we witnessed a consistent temporal behavior, where the maximal number of consecutive time points with abundance greater than 0 is well over 10 (Supplementary Information S5 Fig).

### The autoregressive component of an adult versus infant microbiome

**Fig 5:**
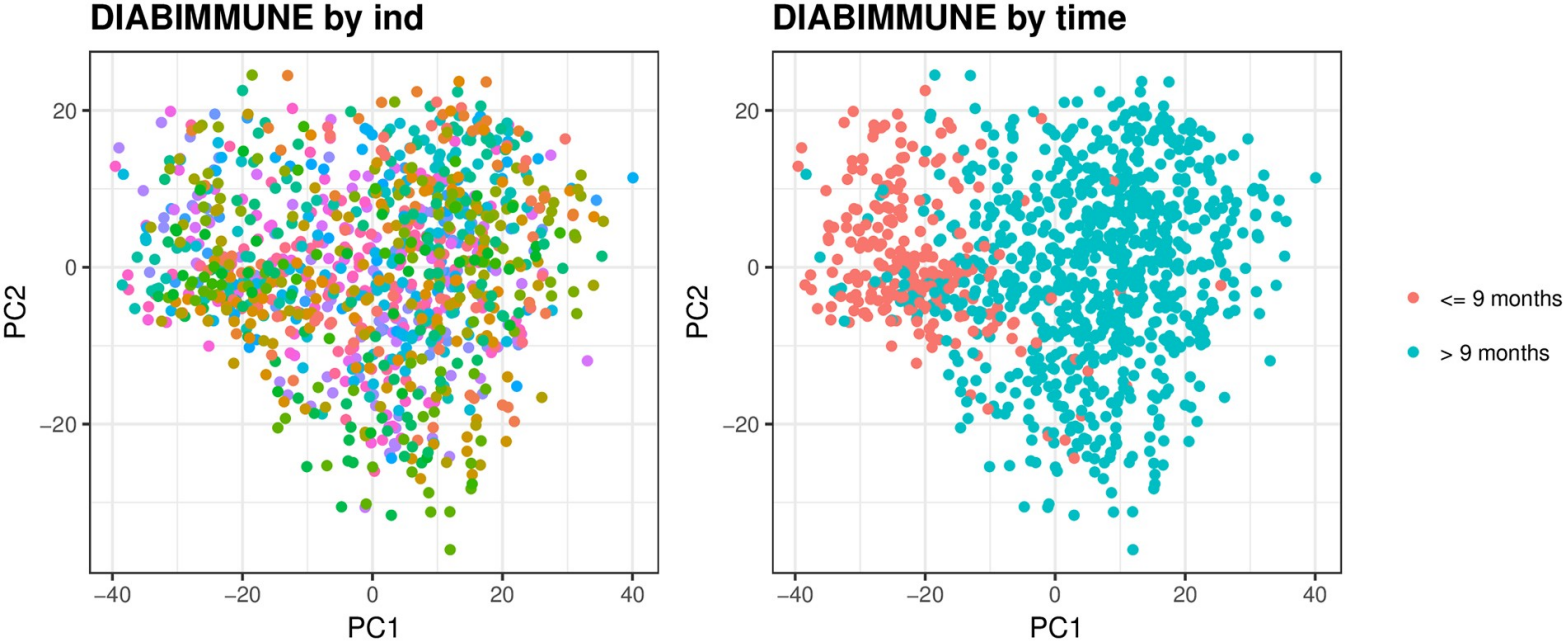
The first two principal components of the temporal kinship matrix in infants. The first two principal components of the temporal kinship matrix color coded by individual (left; 39 infant donors) and by time (right; before and after nine months) using the DIABIMMUNE data.

The colonization of the human gut begins at birth and is characterized by a succession of microbial consortia [35–38], where the diversity and richness of the microbiome reach adult levels in early childhood. A longitudinal study has recently been used to show that infant gut microbiome begins transitioning towards an adult-like community after weaning [39]. This observation is validated using our infant longitudinal data set (DIABIMMUNE) by applying PCA to the temporal kinship matrix (Fig 5). Our analysis reveals that the first principal component (accounting for 26% of the overall variability) is associated with time. Specifically, there is a clear clustering of the time samples from the first nine months of an infant’s life and the rest of the time samples (months 10 − 36) which may be correlated to weaning. As expected, we find a strong autoregressive component in an infant microbiome, which is highly associated with temporal variation across individuals. By applying PCA to the temporal kinship matrix, we demonstrate that there is high similarity in the microbial community composition of infants at least in the first 9 months. This similarity increases the power of our algorithm and thus helps *MTV-LMM* to detect autoregressive taxa.

**Fig 6:**
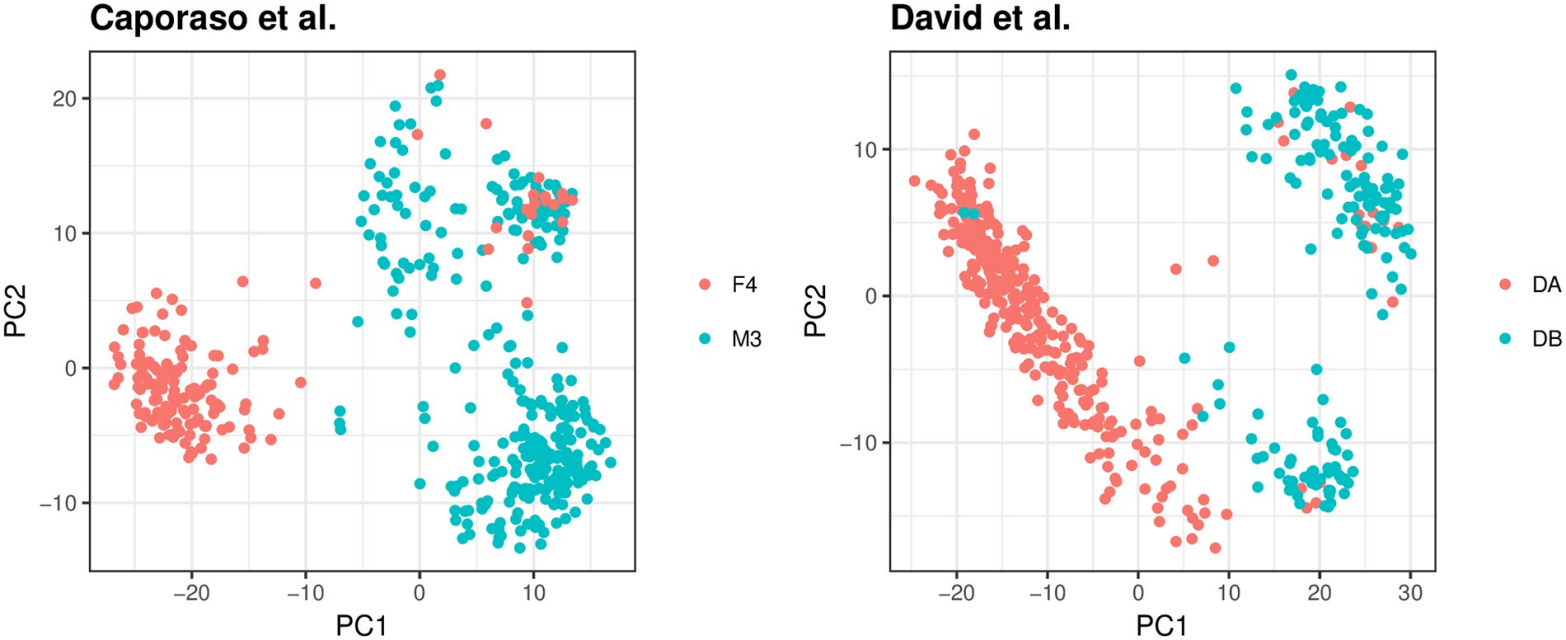
The first two principal components of the temporal kinship matrix in adults. The first two principal components of the temporal kinship matrix color coded by individual. Caporaso et al. [16](left; 2 adult donors: M3, F4) and David et al. [17](right; 2 adult donors: DA, DB).

In contrast to the infant microbiome, the adult microbiome is considered relatively stable [16, 40], but with considerable variation in the constituents of the microbial community between individuals. Specifically, it was previously suggested that each individual adult has a unique gut microbial signature [41–43], which is affected, among others factors, by environmental factors [20] and host lifestyle (i.e., antibiotics consumption, high-fat diets [17] etc.). In addition, [17] showed that over the course of one year, differences between individuals were much larger than variation within individuals. This observation was validated in our adult datasets (David et al. and Caporaso et al.) by applying PCA to the temporal kinship matrices. In both David et al. and Caporaso et al., the first principal component, which accounts for 61% and 43% of the overall variation respectively, is associated with the individual’s identity (Fig 6).

Using *MTV-LMM* we observed that despite the large similarity along time within adult individuals, there is also a non-negligible autoregressive component in the adult microbiome. The fraction of variance explained by time across individuals can range from 6% up to 79% for different taxa. These results shed more light on the temporal behavior of taxa in the adult microbiome, as opposed to that of infants, which are known to be highly affected by time [39].

## Materials and methods

### *MTV-LMM* algorithm

*MTV-LMM* uses a linear mixed model (see [44] for a detailed review), a natural extension of standard linear regression, for the prediction of time series data. We describe the technical details of the linear mixed model below.

We assume that the relative abundance levels of focal taxa *j* at time point *t* depend on a linear combination of the relative abundance levels of the microbial community at previous time points. We further assume that temporal changes in relative abundance levels, in taxa *j*, are a time-homogeneous high-order Markov process. We model the transitions of this Markov process using a linear mixed model, where we fit the *p* previous time points of taxa *j* as fixed effects and the *q* previous time points of the rest of the microbial community as random effects. *p* and *q* are the temporal parameters of the model.

For simplicity of exposition, we present the generative linear mixed model that motivates the approach taken in *MTV-LMM* in two steps. In the first step we model the microbial dynamics in one individual host. In the second step we extend our model to *N* individuals, while accounting for the hosts’ effect.

We first describe the model assuming there is only one individual. Consider a microbial community of *m* taxa measured at *T* equally spaced time points. We get as input an *m* × *T* matrix *M*, where *M*_*jt*_ represents the relative-abundance levels of taxa *j* at time point *t*. Let *y*^*j*^ = (*M*_*j*,*p*+1_, …, *M*_*jT*_)^*t*^ be a (*T* − *p*) × 1 vector of taxa *j* relative abundance, across *T* − *p* time points starting at time point *p* + 1 and ending at time point *T*. Let *X*^*j*^ be a (*T* − *p*) × (*p* + 1) matrix of *p* + 1 covariates, comprised of an intercept vector as well as the first *p* time lags of taxa *j* (i.e., the relative abundance of taxa *j* in the *p* time points prior to the one predicted). Formally, for *k* = 1 we have $X_{tk}^{j}=1$, and for 1 < *k* ≤ *p* + 1 we have $X_{tk}^{j}=M_{j,t-k+1}$ for *t* ≥ *k*. For simplicity of exposition and to minimize the notation complexity, we assume for now that *p* = 1. Let *W* be an (*T* − *q*) × *q* ⋅ *m* normalized relative abundance matrix, representing the first *q* time lags of the microbial community. For simplicity of exposition we describe the model in the case *q* = 1, and then *W*_*tj*_ = *M*_*jt*_ (in the more general case, we have *W*_*tj*_ = *M*_⌈*j*/*q*⌉,*t*−(*jmodq*)_, where *p*, *q* ≤ *T* − 1).

With these notations, we assume the following linear model:

$$y^{j}=X^{j}\beta^{j}+Wu^{j}+\epsilon^{j},$$ (1)

where *u*^*j*^ and *ϵ*^*j*^ are independent random variables distributed as $u^{j}∼N(0_{m},\sigma_{u^{j}}^{2}I_{m})$ and $\epsilon^{j}∼N\left(0_{T-1},\sigma_{\epsilon^{j}}^{2}I_{T-1}\right)$. The parameters of the model are *β*^*j*^ (fixed effects), $\sigma_{u^{j}}^{2}$, and $\sigma_{\epsilon^{j}}^{2}$.

We note that environmental factors known to be correlated with taxa abundance levels (e.g., diet, antibiotic usage [17, 20]) can be added to the model as fixed linear effects (i.e., added to the matrix *X*^*j*^).

Given the high variability in the relative abundance levels, along with our desire to efficiently capture the effects of multiple taxa in the microbial community on each focal taxa *j*, we represent the microbial community input data (matrix *M*) using its quantiles. Intuitively, we would like to capture the information as to whether a taxa is present or absent, or potentially introduce a few levels (i.e., high, medium, and low abundance). To this end, we use the quantiles of each taxa to transform the matrix *M* into a matrix $\tilde{M}$, where ${\tilde{M}}_{jt}\in \left\{0,1,2\right\}$ depending on whether the abundance level is low (below 25% quantile), medium, or high (above 75% quantile). We also tried other normalization strategies, including quantile normalization, which is typically used in gene expression eQTL analysis [45, 46], and the results were qualitatively similar (see Supplementary Information S6 Fig). We subsequently replace the matrix *W* by a matrix $\tilde{W}$, which is constructed analogously to *W*, but using $\tilde{M}$ instead of *M*.

Notably, both the fixed effect (the relative abundance of *y*^*j*^ at previous time points) and the output of *MTV-LMM* are the continuous relative abundance. The random effects are quantile-binned relative abundance of the rest of the microbial community at previous time points (matrix $\tilde{W}$). Thus, our model can now be described as

$$y^{j}=X^{j}\beta^{j}+\tilde{W}u^{j}+\epsilon^{j}$$ (2)

So far, we described the model assuming we have time series data from one individual. We next extend the model to the case where time series data is available from multiple individuals. In this case, we assume that the relative abundance levels of *m* taxa, denoted as the microbial community, have been measured at *T* time points across *N* individuals. We assume the input consists of *N* matrices, *M*^1^, …, *M*^*N*^, where matrix *M*^*i*^ corresponds to individual *i*, and it is of size *m* × *T*. Therefore, the outcome vector *y*^*j*^ is now an *n* × 1 vector, composed of *N* blocks, where *n* = (*T* − 1)*N*, and block *i* corresponds to the time points of individual *i*. Formally, $y_{k}^{j}=M_{j,(k\;mod\;(T-1))}^{\left\lceil k/(T-1)\right\rceil }$. Similarly, we define *X*^*j*^ and $\tilde{W}$ as block matrices, with *N* different blocks, where corresponds to individual *i*.

When applied to multiple individuals, Model (2) may overfit to the individual effects (e.g., due to the host genetics and or environment). In other words, since our goal is to model the changes in time, we need to condition these changes in time on the individual effects, that are unwanted confounders for our purposes. We therefore construct a matrix *H* by randomly permuting the rows of each block matrix *i* in $\tilde{W}$, where the permutation is conducted only within the same individual. Formally, we apply permutation *π*_*i*_ ∈ *S*_*T*−1_ on the rows of each block matrix *i*, *M*^*i*^, corresponding to individual *i*, where *S*_*T*−1_ is the set of all permutations of (*T* − 1) elements. In each *π*_*i*_, we are simultaneously permuting the entire microbial community. Hence, matrix *H* corresponds to the data of each one of the individuals, but with no information about the time (since the data was shuffled across the different time points). With this addition, our final model is given by

$$y^{j}=X^{j}\beta^{j}+\tilde{W}u^{j}+Hr+\epsilon^{j},$$ (3)

where $u^{j}∼N\left(0_{m},\sigma_{u^{j}}^{2}I_{m}\right)$ and $\epsilon^{j}∼N\left(0_{n},\sigma_{\epsilon^{j}}^{2}I_{n}\right)$, and $r∼N(0_{m},\sigma_{r}^{2}I_{m})$. It is easy to verify that an equivalent mathematical representation of model 3 can be given by

$$y^{j}∼N\left(X^{j}\beta^{j},\sigma_{AR^{j}}^{2}K_{1}+\sigma_{ind}^{2}K_{2}+\sigma_{\epsilon^{j}}^{2}I\right),$$ (4)

where $\sigma_{AR^{j}}^{2}=m\sigma_{u^{j}}^{2}$, $K_{1}=\frac{1}{m}\tilde{W}{\tilde{W}}^{T}$, $\sigma_{ind}^{2}=m\sigma_{r}^{2}$, $K_{2}=\frac{1}{m}HH^{T}$. We will refer to *K*_1_ as the *temporal kinship matrix*, which represents the similarity between every pair of samples across time (i.e., represents the cross-correlation structure of the data).

We note that for the simplicity of exposition, we assumed so far that each sample has the same number of time points *T*, however in practice the number of samples may vary between the different individuals. It is easy to extend the above model to the case where individual *i* has *T*_*i*_ time points, however the notations become cumbersome; the implementation of *MTV-LMM*, however takes into account a variable number of time points across the different individuals.

Once the distribution of *y*^*j*^ is specified, one can proceed to estimate the fixed effects *β*^*j*^ and the variance of the random effects using maximum likelihood approaches. One common approach for estimating variance components is known as restricted maximum likelihood (REML). We followed the procedure described in the GCTA software package [47], under ‘GREML analysis’, originally developed for genotype data, and re-purposed it for longitudinal microbiome data. GCTA implements the restricted maximum likelihood method via the average information (AI) algorithm.

Specifically, we performed a restricted maximum likelihood analysis using the function “–reml” followed by the option “–mgrm” (reflects multiple variance components) to estimate the variance explained by the microbial community at previous time points. To predict the random effects by the BLUP (best linear unbiased prediction) method we use “–reml-pred-rand”. This option is actually to predict the total temporal effect (called “breeding value” in animal genetics) of each time point attributed by the aggregated effect of the taxa used to estimate the temporal kinship matrix. In both functions, to represent *y*^*j*^ (the abundance of taxa *j* at the next time point), we use the option “–pheno”. For a detailed description see Supplementary Information S3 Note.

### Time-explainability

We define the term *time-explainability*, denoted as *χ*, to be the temporal variance explained by the microbial community in the previous time points. Formally, for taxa *j* we define

$$\chi^{j}=\frac{\sigma_{AR^{j}}^{2}}{\sigma_{AR^{j}}^{2}+\sigma_{ind}^{2}+\sigma_{\epsilon^{j}}^{2}}$$

The time-explainability was estimated with GCTA, using the temporal kinship matrix. In order to measure the accuracy of time-explainability estimation, the average confidence interval width was estimated by computing the confidence interval widths for all autoregressive taxa and averaging the results. Additionally, we adjust the time-explainability P-values for multiple comparisons using the Benjamini-Hochberg method [48].

### Best linear unbiased predictor

We now turn to the task of predicting $y_{t}^{j}$ using the taxa abundance in time *t* − 1 (or more generally in the last few time points). Using our model notation, we are given *x*^*j*^ and $\tilde{w}$, the covariates associated with a newly observed time point *t* in taxa *j*, and we would like to predict $y_{t}^{j}$ with the greatest possible accuracy. For a simple linear regression model, the answer is simply taking the covariate vector *x* and multiplying it by the estimated coefficients $\hat{\beta }:{\hat{y}}_{t}^{j}=x^{T}\hat{\beta }$. This practice yields unbiased estimates. However, when attempting prediction in the linear mixed model case, things are not so simple. One could adopt the same approach, but since the effects of the random components are not directly estimated, the vector of covariates $\tilde{w}$ will not contribute directly to the predicted value of $y_{t}^{j}$, and will only affect the variance of the prediction, resulting in an unbiased but inefficient estimate. Instead, one can use the correlation between the realized values of $\tilde{W}u$, to attempt a better guess at the realization of $\tilde{w}u$ for the new sample. This is achieved by computing the distribution of the outcome of the new sample conditional on the full dataset, by using the following property of the multivariate normal distribution. Assume we sampled *t* − 1 time points from taxa *j*, but the relative abundance level for the next time point *t*, $y_{t}^{j}$, is held out from the algorithm. The conditional distribution of $y_{t}^{j}$ given the relative abundance levels at all previous time points, *y*^*j*^, is given by:

$$y_{t}^{j}|y^{j}∼N\left(x^{T}\beta^{j}+\Sigma_{t,-t}\Sigma_{-t,-t}^{-1}\left(y^{j}-X^{j}\beta^{j}\right),\Sigma_{t,-t}\Sigma_{-t,-t}^{-1}\Sigma_{-t,t}\right),$$ (5)

where $\Sigma =\tilde{W}{\tilde{W}}^{T}\sigma_{u^{j}}^{2}+HH^{T}\sigma_{r}^{2}+I\sigma_{\epsilon^{j}}^{2}$ and positive/negative indices indicate the extraction/removal of rows or columns, respectively. Intuitively, we use information from the previous time points that have a high correlation with the new time point, to improve its prediction accuracy. The practice of using the conditional distribution is known as BLUP (Best Linear Unbiased Predictor). Therefore, *MTV-LMM* could be used to learn taxa effects in a train set (taxa abundance at time points 1, …, *t*), and subsequently use these learned taxa effects to predict the temporal-community contribution in the next time point in a test set (taxa *j* at *t* + 1). We will define the association matrix *U* (*m* × *m*) using BLUP, where *u*_*ij*_ is the effect of taxa *i* on taxa *j*.

### Prediction accuracy

The predictive ability of a model is commonly assessed using the prediction error variance, $PEV=Var(y^{j}-{\hat{y}}^{j})$, where ${\hat{y}}^{j}$ is the Best Linear Unbiased Predictor of *y*^*j*^. The proportional reduction in relative abundance variance accounted for by the predictions (referred to as *R*^2^ in this paper) can be quantified using

$$R^{2}=\frac{Var\left(y^{j}\right)-Var\left({\hat{y}}^{j}\right)}{Var(y^{j})}=\frac{Cov{\left(y^{j},{\hat{y}}^{j}\right)}^{2}}{Var\left(y^{j}\right)Var\left({\hat{y}}^{j}\right)}$$

Notably, this definition is equivalent to the squared Pearson correlation.

For every *t* ∈ {*p* + 1, ⋯, *T*}, we calculate ${\hat{y}}_{t}^{j}$, where *p* ≥ *q* and the microbial community composition at time *t* was held out from the algorithm. We next compute *R*^2^ between $y_{\left\{p+1,\cdots ,T\right\}}^{j}$ and ${\hat{y}}_{\left\{p+1,\cdots ,T\right\}}^{j}$.

### Model selection

Given that the model presented in Eq (3) can be extended to any arbitrary *p* and *q*, we tested four different variations of this model: 1. *p* = 0 and *q* = 1 (no fixed effect, random effects based on 1-time lag), 2. *p* = 1 and *q* = 1 (one fixed effect based on 1-time lag, random effects based on 1-time lag), 3. *p* = 0 and *q* = 3 (no fixed effect, random effects based on 3-time lags) and 4. *p* = 1 and *q* = 3 (one fixed effect based on 1-time lag, random effects based on 3-time lags). We divide each dataset into three parts—training, validation, and test, where each part is approximately 1/3 of the time series (sequentially). We train all four models presented above and use the validation set to select a model for each taxa *j* based on the highest correlation with the observed relative abundance. We then compute sequential out-of-sample predictions on the test set with the selected model. Based on this metric, we found *p* = 1 and *q* = 1 to be the best model for most taxa. We use these parameters when comparing with the other methods such as sVAR and ARIMA-Poisson.

There are three main justifications for the use of multiple time points in the model. First, Gibbons et al. [24] empirically preformed a time-lag analysis and found that for most taxa the autocorrelation disappeared after 3 or 4 days, whereas for some taxa the autocorrelation disappeared after 1 or 2 days. Second, previous studies [26, 27, 49, 50] found that the human microbiome reaches equilibrium within 10 days following small perturbations to the community. It is imperative to model the different taxa in a manner that will fit their temporal patterns. Third, allowing for the use of multiple previous time points increases flexibility so that the model can select the correct time window required for each taxa.

### Phylogenetic analysis

We performed the following phylogenetic analysis. First, in order to test the hypothesis that both autoregressive and non-autoregressive dynamics carry a taxonomic signal, we fitted a linear mixed model, where the kinship matrix is now the phylogenetic distance between pairs of taxa and the outcomes are the time-explainability measurement for each taxa. Second, in order to test the hypothesis that only non-autoregressive dynamics carry a non-random taxonomic signal, we conducted a permutation test by shuffling the taxonomic order assigned to each taxa—generating new random “orders” using 100, 000 iterations. We counted the number of non-autoregressive orders in each iteration, thereby generating a null distribution, which we then used to calculate an exact P-value for the dataset in each iteration.

### Alpha diversity measures

To measure the alpha diversity, we used Shannon-Wiener index, which is defined as *H* = −∑*p*_*j*_ *ln*(*p*_*j*_), where *p*_*j*_ is the relative abundance of species *j*. Shannon-Wiener index accounts for both abundance and evenness of the species present. Additionally, we computed the ‘effective number of species’ (also known as true diversity), the number of equally-common species required to give a particular value of an index. The ‘effective number of species’ associated with a specific Shannon-Wiener index *a* is equal to *exp*(*a*).

### Preliminary taxa screening according to temporal presence-absence patterns

To calculate the temporal kinship matrix we included taxa using the following criteria. A taxa is present in at least 10% of the time points (removes dominant zero abundance taxa). In the David et al. dataset we included 1051 (out of 2804), in the Caporaso et al. dataset we included 922 (out of 3436) and in the DIABIMMUNE dataset we included 1440 (out of 7244) taxa.

### Methods comparison

We compared *MTV-LMM* to two existing methods: sVAR suggested by [24] and Poisson regression suggested by [28]. In the sVAR method, we followed the procedure described in [24], while running the model and computing the prediction for each individual separately, since it can only handle one individual at a time. We then computed an aggregated prediction accuracy score for each taxa, by averaging the prediction accuracy of each individual. In the Poisson regression method, we followed the procedure described in [28], while running the model for all the individuals simultaneously and calculating prediction accuracy for each taxa. We used the taxa that passed the screening suggested in [28] (eliminating any taxa in the data for which there were a small number (< 6) of average reads per sample). In both models, the training set was 0.67 of the data and the test set was the remaining 0.33 of the data. In both cases we used the code supplied by the authors.

### Datasets

We evaluated the performance of *MTV-LMM* using three real longitudional datasets with 16S rRNA gene sequencing. All data sets are publicly available. The first data set was collected and studied by David et al. (2014) [17] (2 adult donors). The next data set was collected and studied by Caporaso et al. (2011) [16] (2 adult donors). The third data set was collected by the ‘DIABIMMUNE’ project and studied by Yassour et al. (2016) [21] (39 infant donors). In order to compare across studies and reduce technical variance between studies, closed reference OTUs were clustered at 99% identity against the Greengenes database 13_8 [51]. Open reference OTU picking was also run [52], in order to look for non-database OTUs that might contribute substantially to community dynamics. OTU tables were normalized by random sub-sampling to contain 10, 000 reads per sample.

*David et al. (2014)* dataset [17]. Stool samples from 2 healthy American adults were collected (donor A = DA and donor B = DB). DA collected gut microbiota samples between days 0 and 364 of the study (total 311 samples). DB primarily collected gut microbiota samples between study days 0 and 252 (total 180 samples). The V4 region of the 16S ribosomal RNA gene subunit was used to identify bacteria in a culture-independent manner. DNA was amplified using custom barcoded primers and sequenced with paired-end 100 bp reads on an Illumina GAIIx according to a previously published protocol [53]. ‘OTU picking’ and ‘quality control’ were performed essentially as described [17]. In this work, we used the OTUs shared across donors (2, 804 OTUs).

*Caporaso et al. (2011)* dataset [16]. Two healthy American adults, one male (M3) and one female (F4), were sampled daily at three body sites (gut (feces), mouth, and skin (left and right palms)). M3 was sampled for 15 months (total 332 samples) and F4 for 6 months (total 131 samples). Variable region 4 (V4) of 16S rRNA genes present in each community sample were amplified by PCR and subjected to multiplex sequencing on an Illumina Genome Analyzer IIx according to a previously published protocol [53]. ‘OTU picking’ and ‘quality control’ were performed essentially as described [16]. In this work, we used the OTUs shared across donors (3, 436 OTUs).

*DIABIMMUNE* dataset [21]. Monthly stool samples collected from 39 Finnish infants aged 2 to 36 months. To analyze the composition of the microbial communities in this cohort, DNA from stool samples was isolated and amplified and V4 region of the 16S rRNA gene was sequenced. Sequences were sorted into OTUs. 16S rRNA gene sequencing was performed essentially as previously described in [21]. In this work, we used all the OTUs in the sample (7, 244 OTUs).

### Code availability

Code is available in https://github.com/cozygene/MTV-LMM.

## Discussion

We have presented *MTV-LMM*, a flexible and computationally efficient tool, which can be easily adapted by researchers to select the core time-dependent taxa, quantify their temporal effects and predict their future abundance. Using *MTV-LMM* we find that in contrast to previous reports, a considerable portion of microbial taxa in both infants and adults display temporal structure that is predictable using the previous composition of the microbial community. In reaching this conclusion we have adopted a number of concepts common in statistical genetics for use with longitudinal microbiome studies. We introduce concepts such as time-explainability and the temporal kinship matrix, which we believe will be of use to other researchers studying longitudinal microbiota dynamics, through the framework of linear mixed models.

Time-explainability can be informative for selecting autoregressive taxa that are essential to understanding the temporal behavior of the microbiome in longitudinal studies. In particular, such taxa can be used to characterize the temporal trajectories of the microbial community. The temporal kinship matrix can be used to uncover low-rank temporal structure. Specifically, as shown in the Results section (Fig 5), applying PCA to the temporal kinship matrix in the DIABIMMUNE infant dataset revealed a clear clustering of the time samples that separate the first nine months of an infant’s life from the rest of the time samples (10-36 months). Further, we have shown that the association matrix estimated by *MTV-LMM* can be used to uncover global patterns in microbial composition. Using the DIABIMMUNE dataset, we found a strong phylogenetic structure suggesting that closely related species have similar association patterns. Finally, we have demonstrated that *MTV-LMM* significantly outperforms commonly used methods for temporal modeling of the microbiome, both in terms of its prediction accuracy as well as in its ability to identify time-dependent taxa.

Using *MTV-LMM*, we have demonstrated that taxa autoregressiveness is a spectrum where certain taxa are almost entirely determined by the community composition at previous time points, some are somewhat dependent on the previous time points, and others are completely independent of previous time points. We further show that *MTV-LMM* can identify autoregressive taxa in both ‘evolving’ (i.e., infant’s gut) and ‘stable’ (i.e., adult gut) ecosystems. In the former case, i.e., infant gut, the organisms are shifting in abundance over time, which will induce autoregressive dynamics. In this case, where succession is one of the main driving forces, a strong phylogenetic signal is expected. In the latter case, i.e., adult gut, the dynamic is more stationary, with occasional blooms of low-abundance taxa that introduce short-term non-stationary behavior. Notably, the ability of *MTV-LMM* to identify time-dependent taxa in both scenarios (i.e., ‘evolving’ and ‘stable’) can be utilized to find keystone species that may be responsible for the temporal changes observed in different ecosystems.

It is important to note that *MTV-LMM* assumes linear dynamics and is built around an AR(p) type of model. However, we recognize that there are also non-linear dynamics in this ecosystem. Nonetheless, it seems that the linear approximation of these dynamics, using the framework of linear mixed models, is capturing a non-negligible signal, which is consistent with other applications of linear mixed models, such as genetics [47] and methylation data [54]. This is demonstrated using both real and simulated longitudinal data where *MTV-LMM* outperforms methods that directly model these non-linear dynamics. Despite the multiple methodological advancements provided by *MTV-LMM*, future refinements are possible. These include modeling count uncertainty as well as applying different transformations to the data (e.g., arcsine). This will allow *MTV-LMM* to model nonlinear correlations and multiplicative errors while accounting for the compositional nature of the data. The instrumental novelty of our method to predict the temporal behavior of taxa is the statistical power that is gained by leveraging the overall community composition as well as all the individuals in the dataset. This suggests that mutual effects of taxa within the microbial community are of major importance in modulating the microbiome’s behavior over time.

## Supporting information

S1 Fig Estimation errors of *MTV-LMM*, TGP-CODA, sVAR and ARIMA Poisson models. Estimation errors calculated using synthetic data, illustrating realistic dynamics and abundance distribution, with 200 taxa and 70 time points, as suggested by Aijo et al. 2018 [29]. Estimation error is defined to be the Euclidean distance between estimated relative abundance and the true ones per time point (Wilcoxon test P-value *MTV-LMM* vs. TGP-CODA = 0.01501, *MTV-LMM* vs. sVAR P-value = 2.224*e* − 08).
(TIFF)

S2 Fig Time-explainability distribution. Time-explainability distribution in Caporaso et al. dataset. The average time-explainability in this cohort is 0.2 (denoted by a dashed line).
(TIFF)

S3 Fig Prediction accuracy (*R*^2^) as a function of time-explainability. (TIFF)

S4 Fig Time-explainability distribution differ by taxonomic order across datasets. Boxplots illustrate the time-explainability distribution across all datasets. Presented are the top seven orders in the DIABIMMUNE dataset.
(TIF)

S5 Fig Relative abundance of taxa from order Burkholderiales in the DIABIMMUNE dataset, colored by individual. Right hand-side, the autoregressive taxa, taxa with a significant time-explainability component (top and bottom: time-explainability = 0.49, 0.35, 95% CI = [0.4, 0.58], [0.33, 0.36]). Left hand-side are the non-autoregressive taxa.
(TIFF)

S6 Fig Sensitivity analysis of the binning parameters used to normalize microbial abundance. Each boxplot corresponds to the prediction accuracy distribution under different binning parameters, i.e., a 25% lower quantile and a 75% upper quantile compared to 5% and 55%, 15% and 65%, 35% and 85%, and quantile normalization. This analysis was conducted on a simulated microbial community composed of 50 species over 50 time points (data was generated as described in the simulation section).
(TIFF)

S1 Table Predictive accuracy comparison. P-values of the Wilcoxon test comparing the prediction accuracy (*R*^2^) of *MTV-LMM* with the prediction accuracy of the AR(1) model, the sVAR model and the ARIMA (1, 0, 0)-Poisson regression model.
(CSV)

S1 Note Simulation study. (PDF)

S2 Note The relation between *MTV-LMM* and the generalized Lotka-Volterra models. (PDF)

S3 Note Replicability/software. (PDF)
