# Supplementary figures and images for: Modeling the temporal dynamics of the gut microbial community in adults and infants
Source: PLoS Comput Biol. 2019 Jun 27;15(6):e1006960. doi: 10.1371/journal.pcbi.1006960 (PMC6597035; doi:10.1371/journal.pcbi.1006960)

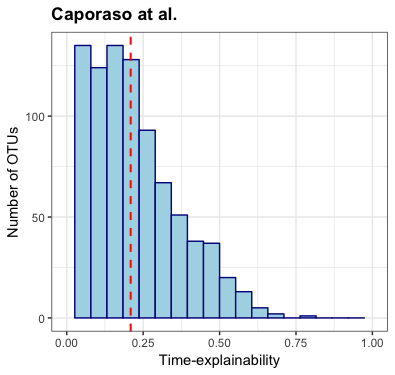

Supplement: S2 Fig — Time-explainability distribution in Caporaso et al. dataset. The average time-explainability in this cohort is 0.2 (denoted by a dashed line). (TIFF) [file pcbi.1006960.s002.tiff]

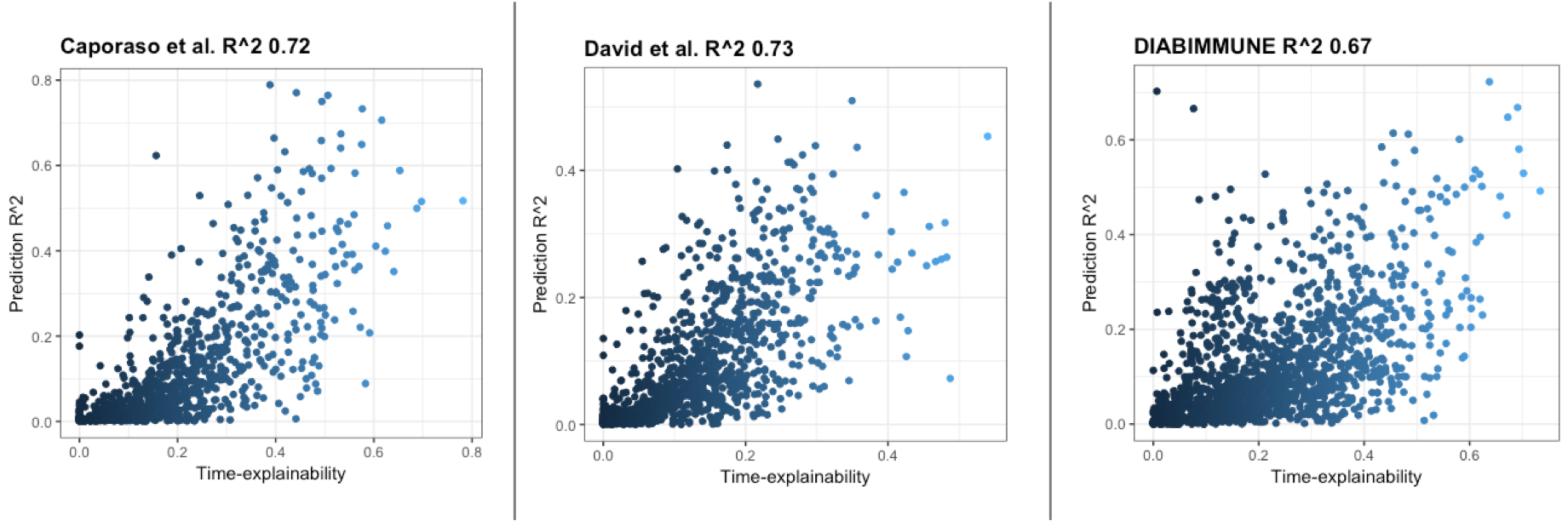

Supplement: S3 Fig — (TIFF) [file pcbi.1006960.s003.tiff]

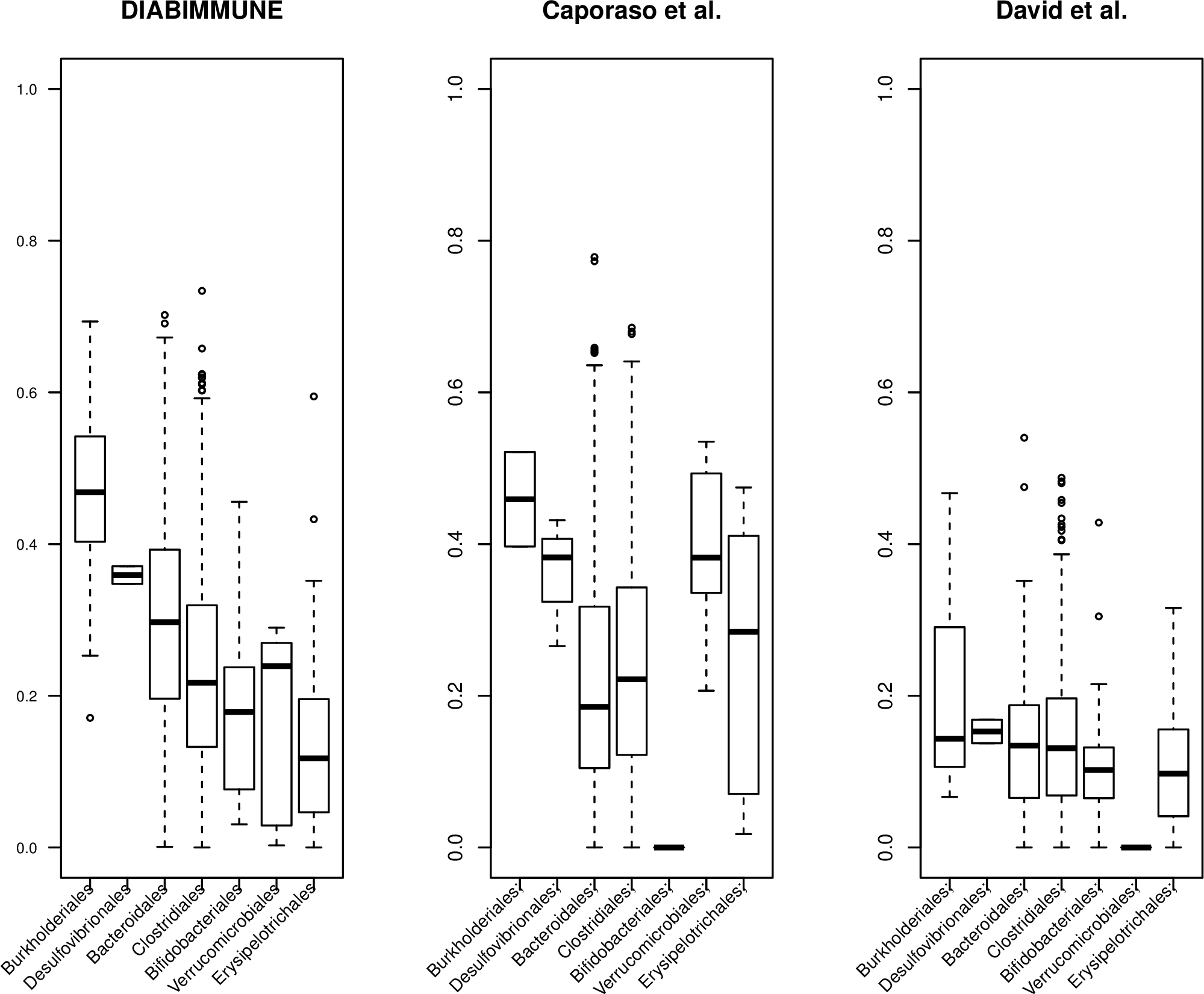

Supplement: S4 Fig — Boxplots illustrate the time-explainability distribution across all datasets. Presented are the top seven orders in the DIABIMMUNE dataset. (TIF) [file pcbi.1006960.s004.tif]

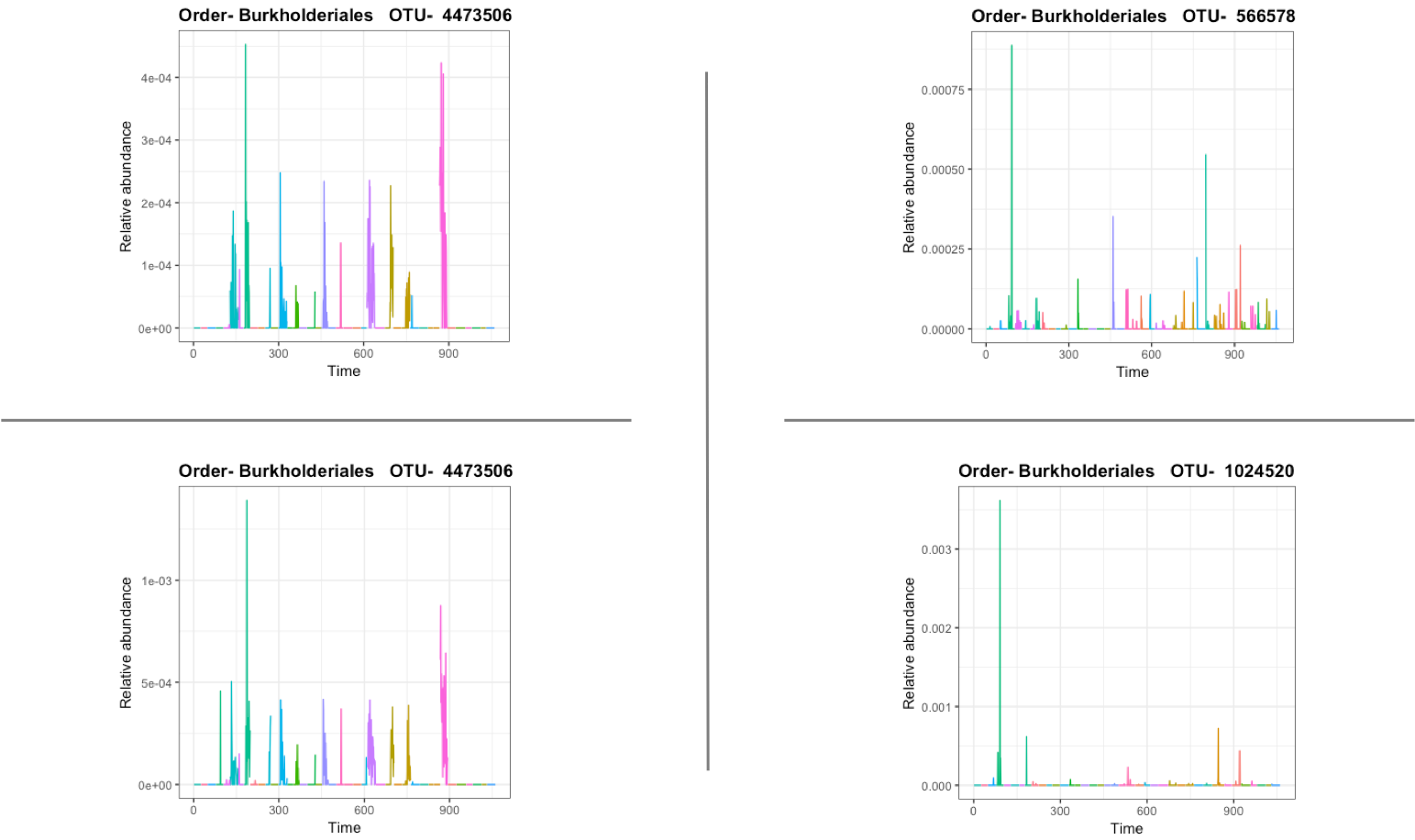

Supplement: S5 Fig — Right hand-side, the autoregressive taxa, taxa with a significant time-explainability component (top and bottom: time-explainability = 0.49, 0.35, 95% CI = [0.4, 0.58], [0.33, 0.36]). Left hand-side are the non-autoregressive taxa. (TIFF) [file pcbi.1006960.s005.tiff]

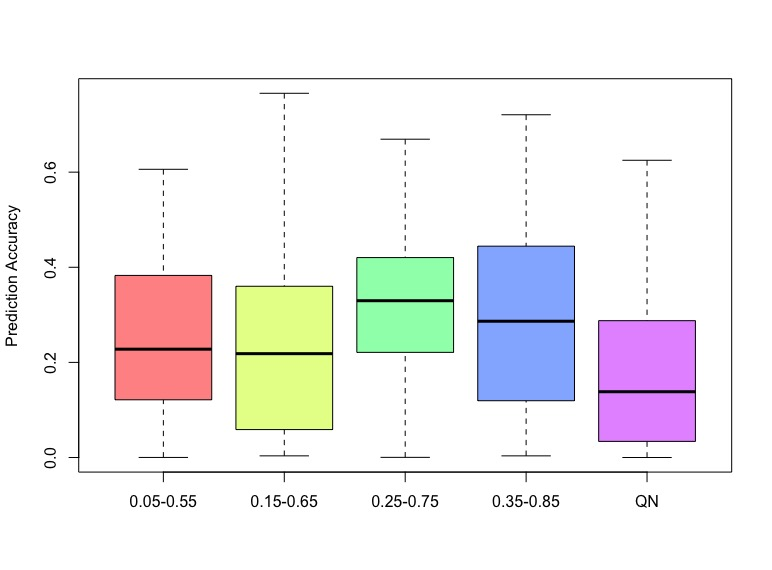

Supplement: S6 Fig — Each boxplot corresponds to the prediction accuracy distribution under different binning parameters, i.e., a 25% lower quantile and a 75% upper quantile compared to 5% and 55%, 15% and 65%, 35% and 85%, and quantile normalization. This analysis was conducted on a simulated microbial community composed of 50 species over 50 time points (data was generated as described in the simulation section). (TIFF) [file pcbi.1006960.s006.tiff]
