# Supplementary material for: Modeling the temporal dynamics of the gut microbial community in adults and infants
Source: PLoS Comput Biol. 2019 Jun 27;15(6):e1006960. doi: 10.1371/journal.pcbi.1006960 (PMC6597035; doi:10.1371/journal.pcbi.1006960)
Supplement: S1 Note — (PDF) [file pcbi.1006960.s008.pdf]

Supplementary Note: Simulation Study To further test the capabilities of *MTV-LMM* to accurately estimate the temporal dynamics and predict the future abundance of microbes within a community, we used synthetic data, illustrating realistic dynamics and abundance distribution, as suggested by Aijo et al. 2018 [1]. Specifically, we consider the Subject A time series from David et al. (2014) [2] and match the relative abundances and dynamics of taxa in synthetic data using real data:

1. We filter the proportion estimates series using a running median filter of length 15; $y_{filt,t} = median(y_{t-7}, y_{t-6}, \ldots, y_{t-1}, y_t, y_{t+1}, y_{t+2}, \ldots, y_{t+6}, y_{t+7})$in order to reduce the amount of noise present in estimates. The filtered estimates are re-normalized to ensure that they sum up to one at each time point.

2. We discard those bacterial species that are lowly abundant (average proportion is less than a threshold) followed by a re-normalization step leaving us noise-free relative abundances of 200 bacterial species.

3. We transform the simplex-valued estimates to real space using the inverse softmax function to add noise and sampling zeros.

4. We add Gaussian distributed noise with zero-mean and standard deviation (SD) $\sigma = 0.5$ and impose a predefined number of sampling zeros by setting corresponding log odds ratios to -10, i.e., to a value that is much smaller than the other values.

5. Noisy relative abundances are obtained by projecting the values onto the simplex using the softmax function.

6. Noisy (overdispersed and zero-inflated) count data (N_t is sampled from the Poisson distribution with the rate $\lambda = 10,000$ are generated from Multinomial distribution using the noisy relative abundances of the part of the Subject A time series (days from 60 to 140) that is highly dynamic David et al. (2014).

Following [1], we evaluate the performance of the model using the 'estimation-error', defined to be the Euclidean distance between estimated relative abundance and the true ones per time point. The 'estimation-error' was calculated on a held-out test set that was kept hidden from the algorithm.

References

1. Tarmo Äijö, Christian L Müller, and Richard Bonneau. Temporal probabilistic modeling of bacterial compositions derived from 16s rrna sequencing. *Bioinformatics*, 34(3):372–380, 2017.

2. Lawrence A David, Arne C Materna, Jonathan Friedman, Maria I Campos-Baptista, Matthew C Blackburn, Allison Perrotta, Susan E Erdman, and Eric J Alm. Host lifestyle affects human microbiota on daily timescales. *Genome biology*, 15(7):R89, 2014.
