# Supplementary material for: Modeling the temporal dynamics of the gut microbial community in adults and infants
Source: PLoS Comput Biol. 2019 Jun 27;15(6):e1006960. doi: 10.1371/journal.pcbi.1006960 (PMC6597035; doi:10.1371/journal.pcbi.1006960)
Supplement: S2 Note — (PDF) [file pcbi.1006960.s009.pdf]

Supplementary Note: The relation between *MTV-LMM* and the generalized Lotka-Volterra models The following serves as a brief introduction to the generalized Lotka-Volterra family of models and its relation to *MTV-LMM*, inspired by the theoretical considerations provided in [1] . The generalized Lotka-Volterra family of models are first-order differential equations that model growth rates as a non-linear function of the community composition, and thus assume the existence of an interaction mechanism between species in the community. *MTV-LMM*, by contrast, assumes linear dynamics. Notably, in both the generalized Lotka-Volterra and *MTV-LMM*, dynamics are defined by species-species interaction terms.

To further elaborate, generalized Lotka-Volterra models are given by:

$$\frac{dy_i}{dt} = a_i y_i(t)\left(1 - \frac{y_i(t)}{K}\right) + y_i(t)\sum_{j=1, j\neq i}^{n} u_{ij} y_j(t)$$

where y_i is the abundance of species i, t is the time , a_i is the self-interaction coefficient for species i, u_{ij} is the interaction coefficient between species i and j, K is abundance and n is the number of species in the community. Dividing by abundance and converting to the difference equation form allows for generalized Lotka-Volterra parameters to be solved with a system of linear equations

$$log(y_i(t)) - log(y_i(t-1)) = a_i - \frac{a_i}{K}\cdot y_i(t-1) + \sum_{j=1, j\neq i}^{n} u_{ij} y_j(t-1)$$

In this form, we can draw similarities to *MTV-LMM* :

$$y_i(t) = \beta_{i_0} + \beta_i y_i(t-1) + \sum_{j=1, j\neq i}^{n} u_{ij} f(y_j(t-1)) + \sum_{j=1, j\neq i}^{n} r_{ij} f(y_{j_{ind}}(t-1)) + \epsilon_i(t)$$

where u_{ij} is the effect of species i on species j, f is a normalization/binning function (e.g quantile normalization) and $\epsilon_i(t)$ is the error term, r are the effects of individual hosts and $y_{j_{ind}}$ corresponds to the data of each one of the individuals, but with no information about the time. Thus, both

generalized Lotka-Volterra and *MTV-LMM* can be solved using linear equations, but with different interpretation to the coefficients. The most significant difference is the fact that *MTV-LMM* assumes a stochastic process (by introducing the error term $\epsilon_i(t)$, while the Lotka-Volterra model implies a fully deterministic model. Other evident differences are that *MTV-LMM* model the observed abundance data, can include any number of time lags (AR(p) process), and the linear function is a function of a transformed version of the abundance levels using quantile-normalization, while generalized Lotka-Volterra models maps the observed abundance data to a difference of the log-transformed data and can only include one time lag, and the linear function is a direct function of the abundance levels.

Notably, *MTV-LMM* has a natural interpretation, similar to the one suggested by the generalized Lotka-Volterra - it assumes that the abundance of species i at time $t+1$ is affected by the abundance levels of many species at time t. The underlying assumption is that each of these effects is small, with a normal prior distribution on the effect size with mean 0 and some variance σ^2. Nonetheless, the generalized Lotka-Volterra does not assume any prior distribution associated with the effects.

A VAR(1) process can be modeled in a similar way:

$$y_i(t) = \beta_{i_0} + \beta_i y_i(t-1) + \sum_{j=1, j\neq i}^{n} u_{ij} y_j(t-1) + \epsilon_i(t)$$

The main methodological advancements of *MTV-LMM*, in comparison to a VAR(p) process, are (1) the ability to model the effects of the microbial community with only one parameter $\sigma^2_{AR^j}$ (as opposed to number of taxa if we only use a model with fixed effects), and (2) the ability to account for the effect of the individual host. These important advancements, coupled with the ability to use multiple variance components, grant *MTV-LMM* its improved scalability both in terms of number of individuals as well as number of taxa which is crucial when studying microbiome data.

References

1. Sean M Gibbons, Sean M Kearney, Chris S Smillie, and Eric J Alm. Two dynamic regimes in the human gut microbiome. *PLoS computational biology*, 13(2):e1005364, 2017.
