# Supplementary material for: Modeling the temporal dynamics of the gut microbial community in adults and infants
Source: PLoS Comput Biol. 2019 Jun 27;15(6):e1006960. doi: 10.1371/journal.pcbi.1006960 (PMC6597035; doi:10.1371/journal.pcbi.1006960)
Supplement: S3 Note — (PDF) [file pcbi.1006960.s010.pdf]

Supplementary Note: Replicability/software

- Identifying auto-regressive taxa by estimating the 'time-explainability' using [1]:
  - Data pre-processing: create the the temporal kinship matrices, fixed effects files and the target files (relative abundance per taxa) - step 1 in *Main-MTV-LMM-TE.R*
  - Model fit: perform a restricted maximum likelihood analysis using the function "–reml" followed by the option "–mgrm" (reflects multiple variance components) using all time points to estimate the variance explained by the microbial community at previous time points - using *run-mgrm.sh*. To represent the abundance of taxa j at the next time point, y^j , we use the option "–pheno".
  - Estimate time-explainability: Calculating the 'time-explainability' for each OTU - step 3 in *Main-MTV-LMM-TE.R*
- Predicting auto-regressive taxa using [1]:
  - Data pre-processing: create the the temporal kinship matrices, fixed effects files and the target files (relative abundance per taxa) - step 1 in *Main-MTV-LMM-TE.R*
  - Prediction: Iteratively perform a restricted maximum likelihood analysis using the function "–reml" followed by the option "–mgrm" (reflects multiple variance components) using time points 1 to t, where $t \in t_{min}, \cdots, t_{max} - 1$ - using $run - predictions.sh$. To represent the abundance of taxa j at the next time point, y^j , we use the option "–pheno".
  - Generating predictions using BLUP - step 3 in *Main-MTV-LMM-Prediction.R*

References

1. Jian Yang, S Hong Lee, Michael E Goddard, and Peter M Visscher. Gcta: a tool for genome-wide complex trait analysis. *The American Journal of Human Genetics*, 88(1):76–82, 2011.
